# GABA_B_-Agonistic Activity of Certain Baclofen Homologues

**DOI:** 10.3390/molecules180910266

**Published:** 2013-08-22

**Authors:** Mohamed I. Attia, Claus Herdeis, Hans Bräuner-Osborne

**Affiliations:** 1 Department of Pharmaceutical Chemistry, College of Pharmacy, King Saud University, P.O. Box 2457, Riyadh 11451, Saudi Arabia; 2 Department of Pharmaceutical Chemistry, Institute of Pharmacy and Food Chemistry, Würzburg University, Am Hubland, Würzburg 97074, Germany; 3 Department of Drug Design and Pharmacology, Faculty of Heath and Medical Sciences, University of Copenhagen, Universitetsparken 2, Copenhagen DK-2100, Denmark

**Keywords:** GABA, synthesis, baclofen homologues, GABA_B_ receptor agonists, pharmacological evaluation

## Abstract

Baclofen (**1**) is a potent and selective agonist for bicuculline-insensitive GABA_B_ receptors and is used clinically as an antispastic and muscle relaxant agent. In the search for new bioactive chemical entities that bind specifically to GABA_B_ receptors, we report here the synthesis of certain baclofen homologues, namely (*R,S*)-5-amino-3-arylpentanoic acid hydrochlorides (*R,S*)-**1a**–**h** as well as (*R,S*)-5-amino-3-methylpentanoic acid [(*RS*)-**1i**] to be evaluated as GABA_B_R agonists. Compound **1a** is an agonist to GABA_B_ receptors with an EC_50_ value of 46 μM on tsA201 cells transfected with GABA_B1b_/GABA_B2_/Gqz5, being the most active congener among all the synthesized compounds.

## 1. Introduction

**Figure 1:**
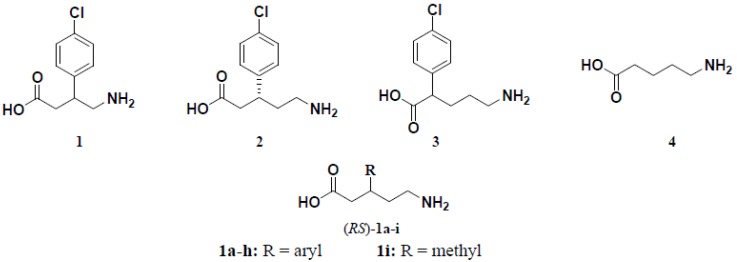
Chemical structures of baclofen (**1**), (*R*)-homobaclofen (**2**), (*RS*)-5-amino-2-(4-chloro- phenyl)pentanoic acid (**3**), 5-aminopentanoic acid (DAVA, **4**) and the target compounds (*RS*)-**1a**–**i**.

4-Aminobutanoic acid (GABA) is the well-known inhibitory neurotransmitter in the mammalian central nervous system where it exerts its effects through ionotropic (GABA_A/C_) receptors and metabotropic (GABA_B_) receptors [1]. Cloning and photoaffinity labeling experiments of the GABA_B_ receptor demonstrated two isoforms, designated GABA_B_1a and GABA_B_1b which dimerize with the GABA_B_2 receptor subunit to produce functionally active GABA_B_ receptors [2]. 4-Amino-3-(4-chlorophenyl)butanoic acid (baclofen, **1**, Figure 1) is the classical GABA_B_ agonist and interacts with GABA_B_ receptors stereospecifically. The GABA_B_ agonistic activity of racemic baclofen is known to reside primarily in the *R*-(-)-enantiomer [3]. (*R,S*)-Baclofen (**1**) is used clinically for the treatment of spasticity associated with brain and spinal cord injuries [4], drug addiction and alcoholism [5], gastroesophageal reflux disease (GERD) [6], cancer pain [7] and overactive bladder [8]. Recently, *R*-(−)-baclofen is under development for the treatment of behavioral symptoms of Fragile X Disorder [9].

(*R*)-5-Amino-3-(4-chlorophenyl)pentanoic acid (**2**), the homologue of baclofen (**1**), has been shown to exhibit a quite remarkable functional pharmacological profile in guinea pig ileum as compared to that of baclofen [10]. On the other hand, the homologue, (*R,S*)-5-amino-2-(4-chlorophenyl)pentanoic acid (**3**), does not interact detectably with GABA_B_ receptors [11]. Moreover, 5-aminopentanoic acid (DAVA, **4**) is a nonselective GABA_B_ antagonist [12]. Using baclofen (**1**) and DAVA (**4**) as two GABA_B_ receptor prototypic ligands, a number of structural hybrids, namely (*R,S*)-5-amino-3-arylpentanoic acid hydrochlorides (*RS*)-**1a**–**h** (Figure 1), containing scaffolds of compounds **2** and **4** were synthesized and pharmacologically characterized as GABA_B_ agonists. The importance of the aromatic moiety on GABA_B_ agonistic activity of compounds (*RS*)-**1a**–**h** was also addressed *via* the synthesis and pharmacological evaluation of their aliphatic analogue, compound **1i**.

## 2. Results and Discussion

### 2.1. Chemistry

An examination of the literature revealed that there are two common synthetic strategies, namely the Horner-Wadsworth-Emmons (HWE) reaction and Knoevenagel condensation, which can be used to prepare the intermediate cyano esters **3a**–**c**, **3e**–**h** and **5i**. Therefore, HWE was applied for preparation of both **3a**–**c** and **3e**–**h** while Knoevenagel condensation was adopted to get **5i**, depending on the commercial availability of their respective starting materials. Accordingly, an allylic bromination step was required jointly with the HWE reaction to prepare compounds **3e**–**h**, while only the HWE reaction and Knoevenagel condensation were required to prepare the cyano esters **3a**–**c** and **5i**, respectively.

**Scheme 1:**
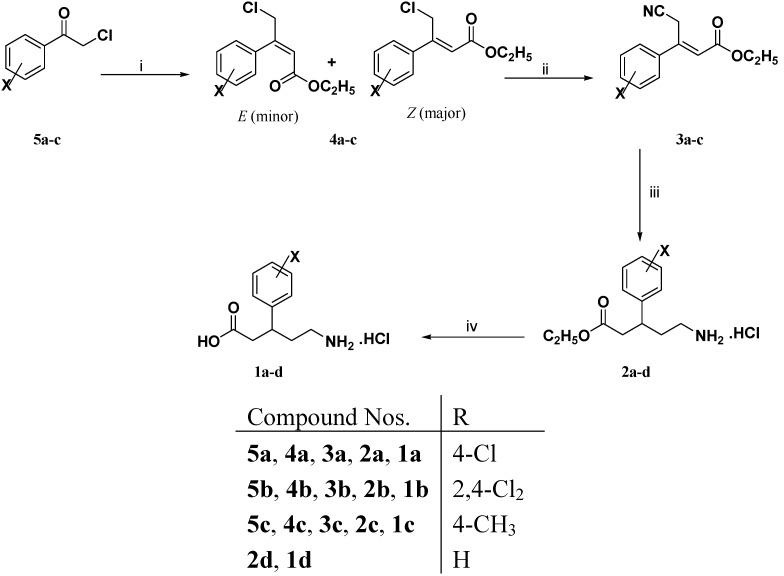
Synthesis of the target compounds **1a**–**d**.

**Scheme 2:**
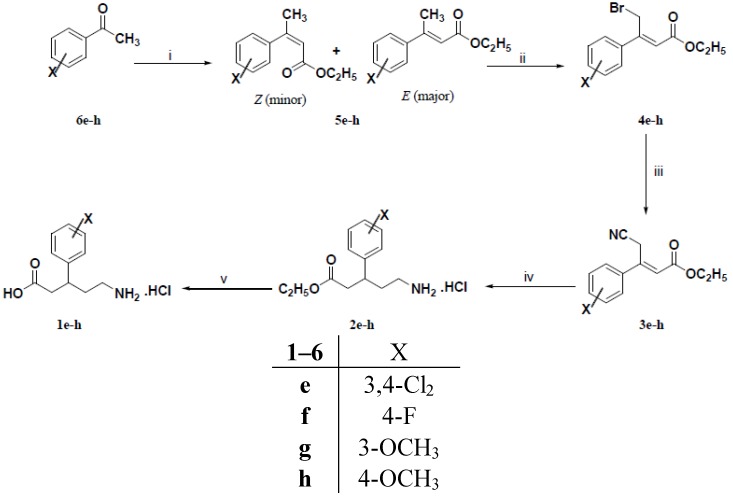
Synthesis of the target compounds **1e**–**h**.

**Scheme 3:**
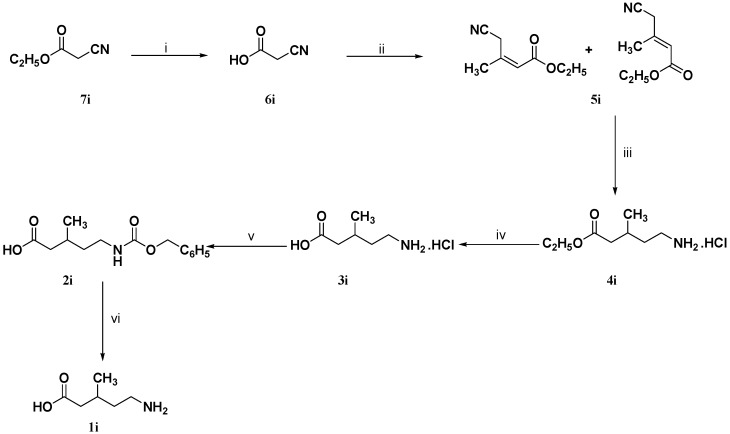
Synthesis of the target compound **1i**.

The synthetic pathways which were adopted to synthesize the target compounds **1a**–**i** are illustrated in Scheme 1, Scheme 2 and Scheme 3. Thus, 3-aryl-4-chloro-2-butenoic acid ethyl esters **4a**–**c** have been successfully produced by applying the HWE reaction on substituted acetophenones **5a**–**c** using triethyl phosphonoacetate and sodium hydride in 1,2 dimethoxyethane following the procedure cited by Wadsworth and Emmons [13] (Scheme 1). The ^13^C-NMR chemical shift differences between C-1, C-3 and in particular C-4 for the (*E*) and (*Z*)-isomers of **4a**–**c** are consistent with the observed differences for (*E*) and (*Z*)-isomers mentioned by Allan and Tran [14].

It is noteworthy that substitution at the *ortho* position of the phenyl ring in 2-chloro-1-(2,4-dichlorophenyl)-1-ethanone (**5b**) increased the proportion of (*E*)-isomer in the produced diasteromeric mixture of 4-chloro-3-(2,4-dichlorophenyl)-2-butenoic acid ethyl ester (**4b**), which is in accordance with the findings of Jones and Maisey [15].

*Reagents and conditions*: (i) (EtO)_2_POCH_2_COOEt/NaH/dry 1,2-dimethoxyethane/50 °C/18 h; (ii) (C_2_H_5_)_4_N CN/CH_3_CN/50 °C/18 h; (iii) H_2_/Pd/C or PtO_2_/4 bar/95% C_2_H_5_OH/conc. HCl /25 °C/18 h; (iv) 5N HCl/reflux/4h.

3-Aryl-4-chloro-2-butenoic acid ethyl esters **4a**–**c** (as diasteromeric mixtures) were subjected to a nucleophilic displacement of the halogen with potassium cyanide in aqueous ethanol to obtain 3-aryl-4-cyano-2-butenoic acid ethyl esters **3a**–**c** via the trivial procedure mentioned by Ives and Sames [16]. Unfortunately, the starting materials decomposed and we did not obtain the anticipated compounds **3a**–**c** in any detectable amounts. This troublesome nucleophilic substitution reaction was successfully achieved using a stoichiometric amount of 3-aryl-4-chloro-2-butenoic acid ethyl esters **4a**–**c** (as diasteromeric mixtures) and tetraethylammonium cyanide (TEAC). The reaction mixture was stirred at 50 °C in acetonitrile for 18 h according to the reported procedure [17]. The crude compounds **3a**–**c** were purified by column chromatography using the appropriate solvent system to afford mainly (*E*)-**3a**–**c** in 42%–66% yields. Use of a catalytic amount of TEAC instead of a stoichiometric amount to produce **3a**–**c** led to a dramatic decrease in the yields.

(*E*)-3-Aryl-4-cyano-2-butenoic acid ethyl esters **3a**–**c** are multifunctional molecules and we aimed to reduce selectively both nitrile and double bond functionalities without affecting the ester functionality to afford the title compounds (*R,S*)-5-amino-3-aryl-pentanoic acid hydrochlorides **1a**–**d**. Catalytic hydrogenation is one of the most powerful methods in the arsenal of the synthetic medicinal chemistry facilitating the chemical synthesis of a myriad of bio-active molecules both in research laboratories and industrial settings. Accordingly, **3a**–**c** were subjected to catalytic hydrogenation using a catalytic amount of PtO_2_ (for **3a** and **3b**) or 10% Pd/C (for **3c**) and concentrated hydrochloric acid in 95% ethanol on a Parr shaker apparatus under 4 bar of H_2_ for 18 h at room temperature to give (*R,S*)-5-amino-3-aryl-pentanoic acid ethyl ester hydrochlorides **2a**–**c**.

It is noteworthy that catalytic hydrogenation of (*E*)-4-cyano-3-(2,4-dichlorophenyl)-2-butenoic acid ethyl ester (**3b**) using 10% Pd/C was accompanied by dehalogenation to give (*R,S*)-5-amino-3-phenylpentanoic acid ethyl ester hydrochloride (**2d**).

Without further purification the ester functionality of (*R,S*)-5-amino-3-arylpentanoic acid ethyl ester hydrochlorides **2a**–**d** was hydrolyzed by refluxing (*R,S*)-**2a**–**d** in 5 N hydrochloric acid for 4 h. The crude (*R,S*)-**1a**–**d** were recrystallized from the isopropanol to afford the target compounds (*R,S*)-**1a**–**d** in 69%–76% yields. The structures of **1a**–**d** have been established through microanalytical, IR, ^1^H- NMR, ^13^C-NMR, and mass spectral data.

Synthesis of the title compounds **1e**–**h** is portrayed in Scheme 2. The synthetic pathway was commenced with the preparation of (*Z*)-3-aryl-4-bromo-2-butenoic acid ethyl esters **4e**–**h**. Chemoselective allylic bromination of 3-aryl-2-butenoic acid ethyl esters **5e**–**h** (as diasteromeric mixtures) was accomplished by adopting Wohl-Ziegler bromination.

*Reagents and conditions*: (i) (EtO)_2_POCH_2_COOEt/KO*t*-Bu/dry THF/reflux/18h; (ii) NBS/benzoyl peroxide/ CCl_4_/reflux/24; (iii) (C_2_H_5_)_4_NCN/CH_3_CN/50 °C/18 h; (iv) H_2_/Pd/C or PtO_2_/4 bar/95% C_2_H_5_OH/conc. HCl/25 °C/18 h; (v) 5N HCl/reflux/4h.

Compounds **5e**–**h** and a stoichiometric amount of *N*-bromosuccinimide (NBS) were refluxed in carbon tetrachloride and then a catalytical amount of dibenzoyl peroxide (DBP) was added to the reaction mixture according to the method advocated by Chiefari *et al.* [18] to afford (*Z*)-3-aryl-4-bromo-2-butenoic acid ethyl esters **4e**–**h** in moderate yields. The isolated isomers of **4e**–**h** were assigned to be (*Z*)-isomers based on their ^1^H-NMR spectral data.

Elaboration of **4e**–**h** to give **3e**–**h** was conducted using the aforementioned procedure for preparation of **3a**–**c**. Subsequently, **3e**–**h** were transformed to the target compounds **1e**–**h** by adopting the same reaction sequence which was previously described for the preparation of compounds **1a**–**d** from **3a**–**c**.

The synthetic plan for the preparation of (*R,S*)-5-amino-3-methylpentanoic acid (**1i**) is provided in Scheme 3. Thus, cyanoacetic acid (**6i**) was subjected to the Knoevenagel reaction using ethyl acetoacetate, ammonium acetate and acetic acid in dry benzene under reflux conditions.

*Reagents and conditions*: (i) 1N HCl/100 °C/1.5 h; (ii) Ethyl acetoacetate/ammonium acetate/acetic acid/benzene/reflux/8 h; (iii) H_2_/Pd/C/conc. HCl/4bar/95% ethanol/25 °C/18 h; (iv) 5N HCl/reflux/4 h/reflux; (v) Benzyl chloroformate/4N NaOH/0 °C/0.5 h; (vi) H_2_/Pd/C/4 bar/50% 2-propanol/25 °C/18 h.

The produced crude 4-cyano-3-methyl-2-butenoic acid ethyl ester (**5i**) was distilled (100–102 °C/5 mm) to afford the α,β-unsaturated diasteromeric mixture **5i** with an *E*/*Z* ratio = 1.7 (lit. [19] = *E*/*Z* ratio = 1.5) as detected by ^1^H-NMR.

4-Cyano-3-methyl-2-butenoic acid ethyl ester (**5i**, as a diasteromeric mixture) was subjected to catalytic hydrogenation using 10% Pd/C and concentrated hydrochloric acid in 95% ethanol to afford (*R,S*)-5-amino-3-methylpentanoic acid ethyl ester hydrochloride (**4i**). Without further purification, the crude **4i** was hydrolyzed by reflux in 5 N hydrochloric acid to give (*R,S*)-5-amino-3-methylpentanoic acid hydrochloride (**3i**). It has to be mentioned that our attempt to obtain compound **3i** in a sufficient pure form by recrystallization was unsuccessful. Accordingly, the amino functionality of **3i** was derivatized with a lipophilic moiety to facilitate its purification by a simple acid-base chemical treatment.

(*RS*)-5-Benzyloxycarbonylamino-3-methylpentanoic acid (**2i**) has been synthesized by adopting the trivial procedure for protecting the amino groups of amino acids [20]. The crude (*R,S*)-5-benzyloxy-carbonylamino-3-methylpentanoic acid (**2i**) was subjected to catalytic hydrogenation to cleave the *N*-benzyloxycarbonyl protecting group. The crude (*R,S*)-5-amino-3-methyl-pentanoic acid (**1i**) was recrystallized from 2-propanol/water to give (*R,S*)-**1i** as a white powder (m.p. 164–165 °C; lit. [21]. 133–135 °C) in 69% yield. The structure of (*R,S*)-**1i** has been established through microanalytical, IR, ^1^H- NMR, ^13^C-NMR, and mass spectral data.

### 2.2. GABA_B_ Agonistic Activity

We have previously described a robust pharmacological assay of heterodimeric GABA_B_R1b/GABA_B_R2 receptors co-expressed with the chimeric G protein Gαq-z5 in tsA201 cells (a transformed HEK293 cell line). Co-expression of Gαq-z5 convert the endogenous coupling to the Gαi/o signaling pathway to the Gq pathway, which generally leads to more robust assays measured as increases in inositol phosphates or intracellular calcium levels [22]. We have previously shown that the pharmacological profiles of a range of standard agonists using this assay correlate well with other assays using either cell lines with recombinant receptor expression or tissues with endogenous GABA_B_R expression. Furthermore, we have shown that the GABA_B_R antagonists 2-OH-saclofen and CGP35348 can antagonize agonist responses in this assay [23,24]. Finally, like other groups [25], we have not found any pharmacological differences of orthosteric ligands between GABA_B_R1a and GABA_B_R1b subunits co-expressed with GABA_B_R2 using this assay [23]. The assay is thus suitable for characterization of orthosteric GABA_B_R ligands, and in the present study we have characterized the synthesized ligands on GABA_B_R1b/GABA_B_R2 receptors co-expressed with the chimeric G protein Gαq-z5 in tsA201 cells measuring responses as increases in intracellular calcium measured by the calcium sensitive fluorescent probe Fluo-4.

**Figure 2:**
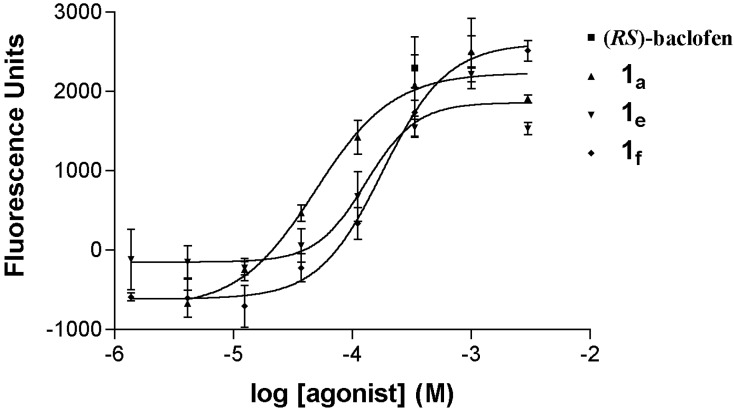
Concentration-response curves of compounds **1a**, **1e**, **1f** and (*RS*)-baclofen on wild type GABA_B_R1b co-expressed with GABA_B_R2 and the chimeric G protein Gαq-z5. The curves are representative for the average pharmacological profile of the agonists. The Ca^2+^ measurement assays were performed as described in the materials and methods section.

The GABA_B_ agonistic activity of the synthesized compounds **1a**–**i** is summarized in Table 1. Compounds **1a**, **1e** and **1f** are active as GABA_B_R agonists (EC_50_ value 46–170 μM, Figure 2) whereas compounds **1b**, **1c**, **1d**, **1g**, **1h** and **1i** (EC_50_ > 300 μM) are considered inactive as GABA_B_R agonists in the GABA_B_R subtype used in our assay.

**Table 1:** GABA_B_ agonistic activity of the target compounds **1a**–**i**. 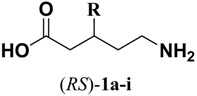

Compound No.	R	EC_50_ (μM)	pEC_50_ ± SEM
**1a**	4-Cl-C_6_H_5_	46	4.34 ± 0.1
**1b**	2,4-Cl_2_-C_6_H_3_	>300	<3.52
**1c**	4-CH_3_-C_6_H_4_	>300	<3.52
**1d**	C_6_H_5_	>300	<3.52
**1e**	3,4-Cl_2_-C_6_H_3_	130	3.89 ± 0.1
**1f**	4-F-C_6_H_4_	170	3.77 ± 0.3
**1g**	3-OCH_3_-C_6_H_4_	>300	<3.52
**1h**	4-OCH_3_-C_6_H_4_	>300	<3.52
**1i**	CH_3_	>300	<3.52
**(*RS*)-baclofen**	-	5.8	5.24 ± 0.1

Regarding the structure-activity relationship in the synthesized series **1a**–**i**, it has to be mentioned that mono-substitution on the aromatic moiety attached to the 3-position of the DAVA backbone with a halogen, especially a *para*-chloro, is optimum for GABA_B_R agonistic activity. The synthesized compounds which evoked GABA_B_R agonistic activity have the following decreasing order of activity: **1a** > **1e** > **1f**. On the other hand, substitution in the *para*-position of the aromatic moiety in the three position of the DAVA backbone with methoxy, methyl or no substitution led to loss of GABA_B_R agonistic activity (EC_50_ > 300 μM). These results are comparable with the previously published results of GABA_B_ agonists [26]. The lack of GABA_B_R agonistic activity of compound **1b** bearing a 2,4-dichloro aromatic moiety in the three position of the DAVA backbone could be attributed to steric reasons which affect the interaction of **1b** with the binding regions of GABA_B_ receptors. In addition, replacement of the aryl moiety in the three position of the DAVA backbone with a methyl group, *i.e*., compound **1i**, led to a loss of GABA_B_R agonistic activity. Compounds **1b**, **1c**, **1d**, **1g**, **1h** and **1i** which showed EC_50_ > 300 μM as GABA_B_R agonists were evaluated as GABA_B_R antagonists at 1 mM concentration against 10 μM GABA, but none of these compounds were effective as GABA_B_R antagonists.

## 3. Experimental

### 3.1. Chemistry

#### 3.1.1. General

Melting points were determined using a capillary melting point apparatus (Gallenkamp, Sanyo) and are uncorrected. Infrared (IR) spectra were recorded as thin layer films (for oils) or as pellets (for solids) with BIO-RAD spectrometer and values are represented in cm^−1^. NMR (^1^H-NMR and ^13^C- NMR) spectra were recorded on a Bruker AC 250 spectrometer (at 250 MHz for ^1^H-NMR and 63 MHz for ^13^C-NMR) and chemical shift values were recorded in ppm on the δ scale. All samples were measured at room temperature. The ^1^H-NMR data are presented as follows: Chemical shifts, multiplicity, number of protons, assignment. Column chromatography was carried out on silica gel 60 (0.063–0.200 mm) obtained from Merck. Elemental analyses were performed by the microanalytical section of the Institute of Inorganic Chemistry, University of Würzburg, Würzburg, Germany.

#### 3.1.2. General Procedure for the Preparation of 3-Aryl-4-chloro-2-butenoic Acid Ethyl Esters **4a–c**

Triethyl phosphonoacetate (2.92 g, 13 mmol) was added dropwise to a cold (5–10 °C) stirred slurry of 60% sodium hydride (0.52 g, 13 mmol) in dry 1,2 dimethoxyethane (20 mL). After complete addition, the reaction mixture was stirred at ambient temperature for 30 min or until gas evolution ceased. A solution of the appropriate ketone **5a**–**c** (10 mmol) in dry 1,2 dimethoxyethane (10 mL) was then added dropwise to the resulting solution. The reaction mixture was heated under stirring at 50 °C for 18 h. The reaction mixture was cooled to room temperature, poured into water (100 mL) and extracted with diethyl ether (3 × 50 mL). The organic extract was dried (Na_2_SO_4_), filtered and evaporated under vacuum to afford viscous oils which were purified by column chromatography using petroleum ether (40–60 °C): Diethyl ether (9:1) to give compounds **4a**–**c** in 40%–88% yields as pale yellow viscous oils.

*(Z)-4-Chloro-3-(4-chlorophenyl)-2-butenoic acid ethyl ester* [(*Z*)-**4a**]. Yield 80%; IR (neat): ν (cm^−1^) = 1711, 1628, 1492, 1176, 1160; ^1^H-NMR (CDCl_3_): δ (ppm) = 1.15 (t, *J* = 7.33 Hz, 3H, CH_3_–CH_2_–), 4.08 (q, *J* = 7.33 Hz, 2H, –CH_2_–CH_3_), 4.88 (s, 2H, 4-H), 6.03 (s, 1H, 2-H), 7.20 (d, *J_AB_* = 8.85 Hz, 2H, H_arom._), 7.30 (d, *J_AB_* = 8.85 Hz, 2H, H_arom._); ^13^C-NMR (CDCl_3_): δ (ppm) = 14.6 (CH_3_–CH_2_–), 39.4 (C-4), 61.1 (–CH_2_–CH_3_), 121.0 (C-2), 128.5, 129.4, 136.2, 137.0 (C_arom._), 151.8 (C-3), 165.7 (C-1).

*(E)-4-Chloro-3-(4-chlorophenyl)-2-butenoic acid ethyl ester* [(*E*)-**4a**]. Yield 8%; IR (neat): ν (cm^−1^) = 1720, 1651, 1491, 1225, 1163; ^1^H-NMR (CDCl_3_): δ (ppm) = 1.16 (t, *J* = 7.03 Hz, 3H, CH_3_–CH_2_–), 4.07 (q, *J* = 7.03 Hz, 2H, –CH_2_–CH_3_), 4.31 (d, *J* = 1.23 Hz, 2H, 4-H), 6.28 (t, *J* = 1.23 Hz, 1H, 2-H), 7.21 (d, *J_AB_* = 8.55 Hz, 2H, H_arom._), 7.39 (d, *J_AB_* = 8.55 Hz, 2H, H_arom._); ^13^C-NMR (CDCl_3_): δ (ppm) = 14.3 (CH_3_–CH_2_–), 48.7 (C-4), 121.5 (C-2), 128.8, 129.5, 135.0, 135.7 (C_arom._), 151.3 (C-3), 165.5 (C-1).

*(Z)-4-Chloro-3-(2,4-dichlorophenyl)-2-butenoic acid ethyl ester* [(*Z*)-**4b**]. Yield 48%; IR (neat): ν (cm^−1^) = 1707, 1641, 1581, 1436, 1341, 1186; ^1^H-NMR (CDCl_3_): δ (ppm) = 1.11 (t, *J* = 7.03 Hz, 3H, CH_3_–CH_2_–), 4.04 (q, *J* = 7.03 Hz, 2H, –CH_2_–CH_3_), 4.79 (s, 2H, 4-H), 5.70 (s, 1H, 2-H), 6.98–7.22 (m, 3H, H_arom._); ^13^C-NMR (CDCl_3_): δ (ppm) = 14.6 (CH_3_–CH_2_–), 40.9 (C-4), 61.3 (–CH_2_–CH_3_), 124.6 (C-2), 127.6, 129.9, 130.0, 132.1, 135.7, 136.9, (C_arom._), 151.9 (C-3), 165.2 (C-1).

*(E)-4-Chloro-3-(2,4-dichlorophenyl)-2-butenoic acid ethyl ester* [(*E*)-**4b**]. Yield 34%; IR (neat): ν (cm^−1^) = 1720, 1585, 1473, 1226, 1164; ^1^H-NMR (CDCl_3_): δ (ppm) = 1.14 (t, *J* = 7.03 Hz, 3H, CH_3_–CH_2_–), 4.06 (q, *J* = 7.03 Hz, 2H, –CH_2_–CH_3_), 4.31 (s, 2H, 4-H), 6.39 (t, *J* = 1.23 Hz, 1H, 2-H), 7.13–7.48 (m, 3H, H_arom._); ^13^C-NMR (CDCl_3_): δ (ppm) = 14.3 (CH_3_–CH_2_–), 47.4 (C-4), 60.9 (–CH_2_–CH_3_), 123.4 (C-2), 127.4, 129.7, 130.9, 132.8, 134.8, 135.2, (C_arom._), 149.3 (C-3), 164.8 (C-1).

*(Z)-4-Chloro-3-(4-methylphenyl)-2-butenoic acid ethyl ester* [(*Z*)-**4c**]. Yield 36%; IR (neat): ν (cm^−1^) = 1710, 1626, 1609, 1173, 1158; ^1^H-NMR (CDCl_3_): δ (ppm) = 1.37 (t, *J* = 7.03 Hz, 3H, CH_3_–CH_2_–), 2.42 (s, 3H, 4–CH_3_), 4.29 (q, *J* = 7.03 Hz, 2H, –CH_2_–CH_3_), 5.12 (s, 2H, 4-H), 6.27 (s, 1H, 2-H), 7.26 (d, *J_AB_* = 8.25 Hz, 2H, H_arom._), 7.50 (d, *J_AB_* = 8.25 Hz, 2H, H_arom._). ^13^C-NMR (CDCl_3_): δ (ppm) = 14.6 (CH_3_–CH_2_–), 21.7 (4–CH_3_), 39.5 (C-4), 60.9 (–CH_2_ –CH_3_), 119.7 (C-2), 127.0, 129.9, 135.6, 140.4, (C_arom._), 153.0 (C-3), 166.0 (C-1).

*(E)-4-Chloro-3-(4-methylphenyl)-2-butenoic acid ethyl ester* [(*E*)-**4c**]. Yield 4%; IR (neat): ν (cm^−1^) = 1703, 1607, 1512, 1225, 1163; ^1^H-NMR (CDCl_3_): δ (ppm) = 1.15 (t, *J* = 7.03 Hz, 3H, CH_3_–CH_2_–), 2.40 (s, 3H, 4–CH_3_), 4.07 (q, *J* = 7.03 Hz, 2H, –CH_2_–CH_3_), 4.33 (d, *J* = 1.23 Hz, 2H, 4-H), 6.26 (t, *J* = 1.23 Hz, 1H, 2-H), 7.16 (d, *J_AB_* = 8.25 Hz, 2H, H_arom._), 7.23 (d, *J_AB_* = 8.25 Hz, 2H, H_arom._); ^13^C-NMR (CDCl_3_): δ (ppm) = 14.4 (CH_3_–CH_2_–), 21.8 (4–CH_3_), 48.9 (C-4), 120.5 (C-2 ), 127.9, 129.3, 134.2, 138.9 (C_arom._), 152.6 (C-3), 165.9 (C-1).

#### 3.1.3. General Procedure for the Preparation of 3-Aryl-2-butenoic Acid Ethyl Esters **5e–h**

To a cold (5–10 °C) solution of potassium *t*-butoxide (1.46 g, 13 mmol) in dry tetrahydrofuran (20 mL) was added dropwise triethyl phosphonoacetate (2.92 g, 13 mmol). The resulting solution was stirred at room temperature for 30 min. A solution of the appropriate ketone **6e**–**h** (10 mmol) in dry tetrahydrofuran (10 mL) was added dropwise to the resulting solution. The reaction mixture was refluxed under stirring for 18 h. The reaction mixture was concentrated under vacuum, diluted with water (100 mL) and extracted with diethyl ether (3 × 50 mL). The combined organic extracts were dried (Na_2_SO_4_), filtered and evaporated under reduced pressure to give viscous oils which were purified by column chromatography using petroleum ether (40–60 °C): Diethyl ether (9:1) to afford compounds **5e**–**h** in 75%–91% yields as pale yellow viscous oils.

*(E)-3-(3,4-Dichlorophenyl)-2-butenoic acid ethyl ester* [(*E*)**-5e**]. Yield 78%; IR (neat): ν (cm^−1^) = 1711, 1630, 1469, 1277, 1169; ^1^H-NMR (CDCl_3_): δ (ppm) = 1.21 (t, *J* = 7.03 Hz, 3H, CH_3_–CH_2_–), 2.42 (d, *J* = 1.23 Hz, 3H, 4-H), 4.12 (q, *J* = 7.03 Hz, 2H, –CH_2_–CH_3_), 5.99 (q, *J* = 1.23 Hz, 1H, 2-H), 7.10–7.44 (m, 3H, H_arom._); ^13^C-NMR (CDCl_3_): δ (ppm) = 14.7 (CH_3_–CH_2_–), 18.1 (C-4), 60.5 (–CH_2_–CH_3_), 118.8 (C-2), 125.9, 128.7, 130.8, 133.2, 133.4, 142.5 (C_arom._), 152.9 (C-3), 166.7 (C-1).

*(Z)-3-(3,4-Dichlorophenyl)-2-butenoic acid ethyl ester* [(*Z*)-**5e**]. Yield 6%; IR (neat): ν (cm^−1^) = 1717, 1644, 1472, 1229, 1165; ^1^H-NMR (CDCl_3_): δ (ppm) = 1.16 (t, *J* = 7.00 Hz, 3H, CH_3_–CH_2_–), 2.17 (d, *J* = 1.53 Hz, 3H, 4-H), 4.07 (q, *J* = 7.00 Hz, 2H, –CH_2_–CH_3_), 5.96 (q, *J* = 1.53 Hz, 1H, 2-H), 7.05–7.46 (m, 3H, H_arom._). ^13^C-NMR (CDCl_3_): δ (ppm) = 14.4 (CH_3_–CH_2_–), 27.3 (C-4), 60.5 (CH_2_–CH_3_), 119.4 (C-2), 126.9, 129.3, 130.3, 132.1, 132.5, 141.1 (C_arom._), 152.9 (C-3) 165.8 (C-1).

*(E)-3-(4-Fluorophenyl)-2-butenoic acid ethyl ester* [(*E*)-**5f**] [27]. Yield 69%; IR (neat): ν (cm^−1^) = 1710, 1631, 1602, 1508, 1233, 1157; ^1^H-NMR (CDCl_3_): δ (ppm) = 1.32 (t, *J* = 7.03 Hz, 3H, CH_3_–CH_2_–), 2.57 (d, *J* = 1.23 Hz, 3H, 4-H), 4.22 (q, *J* = 7.03 Hz, 2H, –CH_2_–CH_3_), 6.10 (q, *J* = 1.23 Hz, 1H, 2-H), 7.02–7.11 (m, 2H, H_arom._), 7.43–7.49 (m, 2H, H_arom._). ^13^C-NMR (CDCl_3_): δ (ppm) = 14.7 (CH_3_–CH_2_–), 18.3 (C-4), 60.3 (CH_2_–CH_3_), 115.8 (d, *J_C-3`, F& C-5`, F_* = 21.99 Hz, C-3` and C-5`), 117.5 (C-2), 128.5 (d, *J_C-2`, F& C-6`,F_* = 7.64 Hz, C-2` and C-6`), 138.6 (d, *J_C-1`, F_* = 2.87 Hz, C-1`), 154.6 (C-3), 163.6 (d, *J_C-4`, F_* = 249.45 Hz, C-4`), 167.1 (C-1).

*(Z)-3-(4-Fluorophenyl)-2-butenoic acid ethyl ester* [(*Z*)-**5f**]. Yield 10%; IR (neat): ν (cm^−1^) = 1718, 1638, 1603, 1509, 1226, 1153; ^1^H-NMR (CDCl_3_): δ (ppm) = 1.00 (t, *J* = 7.00 Hz, 3H, CH_3_–CH_2_–), 2.05 (d, *J*= 1.53 Hz, 3H, 4-H), 3.89 (q, *J* = 7.00 Hz, 2H, –CH_2_–CH_3_), 5.79 (q, *J* = 1.53 Hz, 1H, 2-H), 6.86–6.97 (m, 2H, H_arom._ ), 7.04–7.12 (m, 2H, H_arom._). ^13^C-NMR (CDCl_3_): δ (ppm) = 14.4 (CH_3_–CH_2_–), 27.6 (C-4), 60.2 (–CH_2_–CH_3_), 115.3 (d, *J_C-3`, F& C-5`, F_* = 21.98 Hz, C-3`and C-5`), 118.5 (C-2), 129.2 (d, *J_C-2`, F& C-6`, F_* = 7.60 Hz, C-2` and C-6`), 137.0 (d, *J_C-1`, F_* = 3.82 Hz, C-1`), 154.7 (C-3), 162.8 (d, *J_C-4`, F_* = 247.41 Hz, C-4`), 166.2 (C-1).

*(E)-3-(3-Methoxyphenyl)-2-butenoic acid ethyl ester* [(*E*)-**5g**] [28]. Yield 82%; IR (neat): ν (cm^−1^) = 1709, 1627, 1578, 1216, 1156; ^1^H-NMR (CDCl_3_): δ (ppm) = 1.35 (t, *J* = 7.03 Hz, 3H, CH_3_–CH_2_–), 2.59 (d, *J* = 1.23 Hz, 3H, 4-H), 3.85 (s, 3H, OCH_3_), 4.25 (q, *J* = 7.03 Hz, 2H, –CH_2_–CH_3_), 6.16 (q, *J* = 1.23 Hz, 1H, 2-H), 6.19–7.34 (m, 4H, H_arom._). ^13^C-NMR (CDCl_3_): δ (ppm) = 14.7 (CH_3_–CH_2_–), 18.4 (C-4), 55.7 (OCH_3_), 60.3 (–CH_2_–CH_3_), 112.5 (C-2), 114.7, 117.7, 119.2, 129.9, 144.2 (C_arom._), 155.8 (C-3), 160.0 (C_arom._ ), 167.2 (C-1).

*(Z)-3-(3-Methoxyphenyl)-2-butenoic acid ethyl ester* [(*Z*)-**5g**]. Yield 9%; IR (neat): ν (cm^−1^) = 1724, 1599, 1578, 1213, 1151; ^1^H-NMR (CDCl_3_): δ (ppm) = 1.13 (t, *J* = 7.00 Hz, 3H, CH_3_–CH_2_–), 2.20 (d, *J* = 1.53 Hz, 3H, 4-H), 3.83 (s, 3H, OCH_3_), 4.04 (q, *J* = 7.00 Hz, 2H, –CH_2_–CH_3_), 5.93 (q, *J* = 1.53 Hz, 1H, 2-H), 6.77–7.33 (m, 4H, H_arom._). ^13^C-NMR (CDCl_3_): δ (ppm) = 14.4 (CH_3_–CH_2_–), 27.5 (C-4), 55.6 (OCH_3_), 60.2 (–CH_2_–CH_3_), 113.1 (C-2), 113.4, 118.3, 119.7, 129.4, 142.7 (C_arom._), 155.3 (C-3), 159.6 (C_arom._), 166.3 (C-1).

*(E)-3-(4-Methoxyphenyl)-2-butenoic acid ethyl ester* [(*E*)-**5h**] [29]. Yield 71%; IR (neat): ν (cm^−1^) = 1707, 1603, 1512, 1250, 1153; ^1^H-NMR (CDCl_3_): δ (ppm) = 1.34 (t, *J* = 7.03 Hz, 3H, CH_3_–CH_2_–), 2.59 (d, *J* =1.23 Hz, 3H, 4-H), 3.84 (s, 3H, OCH_3_), 4.23 (q, *J* = 7.03 Hz, 2H, –CH_2_–CH_3_), 6.14 (q, *J* = 1.23 Hz, 1H, 2-H), 6.91 (d, *J_AB_* = 8.85 Hz, 2H, H_arom._), 7.48 (d, *J_AB_* = 8.85 Hz, 2H, H_arom._). ^13^C-NMR (CDCl_3_): δ (ppm) = 14.8 (CH_3_–CH_2_–), 18.0 (C-4), 55.7 (OCH_3_), 60.1 (–CH_2_–CH_3_), 114.2 (C_arom._), 115.7 (C-2), 128.1, 134.7 (C_arom._), 155.2 (C-3), 160.8 (C_arom._), 167.5 (C-1).

*(Z)-3-(4-Methoxyphenyl)-2-butenoic acid ethyl ester* [(*Z*)-**5h**]. Yield 4%; IR (neat): ν (cm^−1^) = 1711, 1606, 1511, 1229, 1156; ^1^H-NMR (CDCl_3_): δ (ppm) = 1.17 (t, *J* = 7.00 Hz, 3H, CH_3_–CH_2_– ), 2.20 (d, *J* = 1.53 Hz, 3H, 4-H), 3.84 (s, 3H, OCH_3_), 4.07 (q, *J* = 7.00 Hz, 2H, –CH_2_–CH_3_), 5.91 (q, *J* = 1.53 Hz, 1H, 2-H), 6.91 (d, *J_AB_* = 8.85 Hz, 2H, H_arom._), 7.23 (d, *J_AB_* = 8.85 Hz, 2H, H_arom._); ^13^C-NMR (CDCl_3_): δ (ppm) = 14.5 (CH_3_–CH_2_–), 27.5 (C-4), 55.6 (OCH_3_), 60.1 (–CH_2_–CH_3_), 113.6 (C_arom._), 117.5 (C-2), 128.9, 133.1 (C_arom._), 155.3 (C-3), 159.8 (C_arom._), 166.5 (C-1).

#### 3.1.4. General Procedure for the Preparation of (*Z*)-3-Aryl-4-bromo-2-butenoic Acid Ethyl Esters **4e–h**

A mixture of 3-aryl-2-butenoic acid ethyl esters **5e**–**h** (9 mmol) and *N*-bromosuccinimide (1.69 g, 10 mmol) was refluxed with stirring. Benzoyl peroxide (0.02 g) was added to the reaction mixture and refluxing was continued for further 24 h. The reaction mixture was chilled and the solid succinimide was filtered off. The filtrate was dried (Na_2_SO_4_), filtered and evaporated under reduced pressure to give viscous oils which were purified by column chromatography using petroleum ether (40–60 °C): Diethyl ether (9:1) to yield mainly (*Z*)-3-aryl-4-bromo-2-butenoic acid ethyl esters **4e**–**h** in 59%–71% yields as light brown viscous oils.

*(Z)-4-Bromo-3-(3,4-dichlorophenyl)-2-butenoic acid ethyl ester* [(*Z*)-**4e**]. Yield 59% as light brown viscous oil; IR (neat): ν (cm^−1^) = 1711, 1626, 1474, 1290, 1178; ^1^H-NMR (CDCl_3_): δ (ppm) = 1.36 (t, *J* = 7.03 Hz, 3H, CH_3_–CH_2_–), 4.29 (q, *J* = 7.03 Hz, 2H, –CH_2_–CH_3_), 4.93 (s, 2H, 4-H), 6.19 (s, 1H, 2-H), 7.38–7.65 (m, 3H, H_arom._); ^13^C-NMR (CDCl_3_): δ (ppm) = 14.6 (CH_3_–CH_2_–), 26.3 (C-4), 61.2 (–CH_2_–CH_3_), 121.4 (C-2), 126.3, 129.0, 131.2, 133.6, 134.3, 138.9 (C_arom._), 151.1 (C-3), 165.5 (C-1).

*(Z)-4-Bromo-3-(4-fluorophenyl)-2-butenoic acid ethyl ester* [(*Z*)-**4f**] [27]. Yield 67% as light brown viscous oil; IR (neat): ν (cm^−1^) = 1709, 1626, 1610, 1510, 1234, 1162; ^1^H-NMR (CDCl_3_): δ (ppm) = 1.36 (t, *J* = 7.03 Hz, 3H, CH_3_–CH_2_–), 4.29 (q, *J* = 7.03 Hz, 2H, –CH_2_–CH_3_), 4.98 (s, 2H, 4-H), 6.19 (s, 1H, 2-H), 7.04–7.17 (m, 2H, H_arom._), 7.52–7.60 (m, 2H, H_arom._); ^13^C-NMR (CDCl_3_): δ (ppm) = 14.6 (CH_3_–CH_2_–), 26.9 (C-4), 61.0 (–CH_2_–CH_3_), 116.3 (d, *J_C-3`, F& C-5`, F_* = 21.57 Hz, C-3`and C-5`), 120.1 (C-2), 129.0 (d, *J_C-2`,__F&__C-6`, F_* = 8.30 Hz, C-2` and C-6`), 134.9 (d, *J_C-1`, F_* = 3.43 Hz, C-1`), 152.5 (C-3), 163.9 (d, *J_C-4`, F_* = 239.36 Hz, C-4`), 165.9 (C-1).

*(Z)-4-Bromo-3-(3-methoxyphenyl)-2-butenoic acid ethyl* *ester* [(*Z*)-**4g**] [30]. Yield 73% as light brown viscous oil; IR (neat): ν (cm^−^^1^) = 1709, 1625, 1579, 1224, 1161; ^1^H-NMR (CDCl_3_): δ (ppm) = 1.37 (t, *J* = 7.00 Hz, 3H, CH_3_–CH_2_– ), 3.87 (s, 3H, OCH_3_), 4.27 (q, *J* = 7.00 Hz, 2H, –CH_2_–CH_3_), 4.98 (s, 2H, 4-H), 6.23 (s, 1H, 2-H), 6.96–7.39 (m, 4H, H_arom._). ^13^C-NMR (CDCl_3_): δ (ppm) = 14.6 (CH_3_–CH_2_–), 27.1 (C-4), 55.8 (OCH_3_), 60.9 (–CH_2_–CH_3_), 112.9 (C-2), 115.5, 119.4, 120.4, 130.2, 140.4 (C_arom._), 153.5 (C-3), 160.2 (C_arom._ ), 165.9 (C-1).

*(Z)-4-Bromo-3-(4-methoxyphenyl)-2-butenoic acid ethyl ester* [(*Z*)-**4h**] [31]. Yield 71% as pale yellow solid m.p. 80–82 °C; IR (neat): ν (cm^−1^) = 1701, 1603, 1512, 1250, 1169; ^1^H NMR (CDCl_3_): δ (ppm) = 1.36 (t, *J* = 7.03 Hz, 3H, CH_3_–CH_2_–), 3.86 (s, 3H, OCH_3_), 4.28 (q, *J* = 7.03 Hz, 2H, –CH_2_–CH_3_), 5.01 (s, 2H, 4-H), 6.21 (s, 1H, 2-H), 6.96 (d, *J_AB_* = 9.15 Hz, 2H, H_arom._), 7.55 (d, *J_AB_* = 9.15 Hz, 2H, H_arom._). ^13^C-NMR (CDCl_3_): δ (ppm) = 14.7 (CH_3_–CH_2_–), 26.8 (C-4), 55.8 (OCH_3_), 60.8 (–CH_2_–CH_3_), 118.1 (C-2), 114.6, 128.4, 130.8, 161.4 (C_arom._), 152.3 (C-3), 166.2 (C-1).

#### 3.1.5. General Procedure for the Preparation of (*E*)-3-Aryl-4-cyano-2-butenoic Acid Ethyl Esters **3a–c** and **3e–h**

A solution of tetraethylammonium cyanide (0.78 g, 5 mmol) in acetonitrile (5 mL) was added dropwise to a stirred solution of 3-aryl-4-chloro-2-butenoic acid ethyl esters **4a**–**c** and/or (*Z*)-3- aryl-4-bromo-2-butenoic acid ethyl esters **4e**–**h** (5 mmol) in acetonitrile (10 mL) under nitrogen atmosphere. After complete addition, the reaction mixture was heated at 50 °C for 18 h. The reaction mixture was cooled, diluted with diethyl ether (30 mL) and washed with water (3 × 20 mL). The organic layer was dried (Na_2_SO_4_) and evaporated under reduced pressure to give dark red viscous oils which were purified by column chromatography using petroleum ether (40–60 °C): Diethyl ether (8:2) to afford mainly (*E*)–3-aryl-4-cyano-2-butenoic acid ethyl esters **3a**–**c** and/or **3e**–**h** as pale yellow viscous oils in 42%–66% yields

*(E)-4-Cyano-3-(4-chlorophenyl)-2-buenoic acid ethyl ester* [(*E*)-**3a**] [32]. Yield 42%; IR (neat): ν (cm^−1^) = 2217, 1731, 1591, 1493, 1176, 1162; ^1^H-NMR (CDCl_3_): δ (ppm) = 1.21 (t, *J* = 7.03 Hz, 3H, CH_3_–CH_2_–), 3.88 ( s, 2H, 4-H), 4.15 (q. *J* = 7.03 Hz, 2H, –CH_2_–CH_3_), 5.79 (s, 1H, 2-H), 7.39 (s, 4H, H_arom._). ^13^C-NMR (CDCl_3_): δ (ppm) = 14.4 (CH_3_–CH_2_–), 39.7 (C-4), 62.0 (CH_2_–CH_3_), 99.9 (C-2), 116.9 (C≡N), 127.9, 129.9, 135.7, 137.1 (C_arom._), 154.9 (C-3), 168.8 (C-1).

*(E)-4-Cyano-3-(2,4-dichloro-phenyl)-2-butenoic acid ethyl ester* [(*E*)-**3b**]. Yield 46%; IR (neat): ν (cm^−1^) = 2223, 1733, 1585, 1472, 1180; ^1^H-NMR (CDCl_3_): δ (ppm) = 1.26 (t, *J* = 7.03 Hz, 3H, CH_3_–CH_2_–), 3.93 (s, 2H, 4-H), 4.16 (q, *J* = 7.03 Hz, 2H, –CH_2_–CH_3_), 5.62 (s, 1H, 2-H), 7.26–7.49 (m, 3H, H_arom._); ^13^C-NMR (CDCl_3_): δ (ppm) = 14.4 (CH_3_–CH_2_–), 40.6 (C-4), 61.9 (–CH_2_–CH_3_), 105.2 (C-2), 115.8 (C≡N), 127.9, 130.3, 131.8, 132.8, 134.4, 136.1 (C_arom._), 155.4 (C-3), 168.4 (C-1).

*(E)-4-Cyano-3-(4-methyl-phenyl)-2-butenoic acid ethyl ester* [(*E*)-**3c**]. Yield 66%; IR (neat): ν (cm^−1^) = 2214, 1733, 1603, 1314, 1175, 1159; ^1^H-NMR (CDCl_3_): δ (ppm) = 1.22 (t, *J* = 7.03 Hz, 3H, CH_3_–CH_2_–), 2.40 (s, 3H, 4`-CH_3_), 3.90 (s, 2H, 4-H), 4.15 (q, *J* = 7.03 Hz, 2H, –CH_2_–CH_3_), 5.78 (s, 1H, 2-H), 7.23 (d, *J_AB_* = 8.23 Hz, 2H, H_arom._), 7.38 (d, *J_AB_* = 8.23 Hz, 2H, H_arom._). ^13^C-NMR (CDCl_3_): δ (ppm) = 14.4 (CH_3_–CH_2_–), 21.7 (4`-CH_3_)_,_ 39.7 (C-4), 61.9 (–CH_2_–CH_3_), 98.3 (C-2), 117.5 (C≡N), 126.4, 130.1, 134.3, 141.4 (C_arom._), 155.9 (C-3), 169.1 (C-1).

*(E)-4-Cyano-3-(3,4-dichlorophenyl)-2-butenoic acid ethyl ester* [(*E*)-**3e**]. Yield 44%; IR (neat): ν (cm^−1^) = 2219, 1732, 1550, 1472, 1179; ^1^H-NMR (CDCl_3_): δ (ppm) = 1.05 (t, *J* = 7.03 Hz, 3H, CH_3_–CH_2_–), 3.68 (s, 2H, 4-H), 3.98 (q, *J* = 7.03 Hz, 2H, –CH_2_–CH_3_), 5.61 (s, 1H, 2-H), 7.08–7.37 (m, 3H, H_arom._); ^13^C-NMR (CDCl_3_): δ (ppm) = 14.4 (CH_3_–CH_2_–), 39.6 (C-4), 62.2 (–CH_2_–CH_3_), 101.1 (C-2), 116.5 (C≡N), 125.8, 128.5, 131.4, 133.9, 135.2, 137.3, (C_arom._),153.9 (C-3), 168.5 (C-1).

*(E)-4-Cyano-3-(4-fluorophenyl)-2-butenoic acid ethyl ester* [(*E*)-**3f**]. Yield 48%; IR (neat): ν (cm^−1^) = 2217, 1732, 1601, 1511, 1237, 1162; ^1^H-NMR (CDCl_3_): δ (ppm) = 1.22 (t, *J* = 7.00 Hz, 3H, CH_3_–CH_2_–), 3.89 (s, 2H, 4-H), 4.16 (q, *J* = 7.00 Hz, 2H, –CH_2_–CH_3_), 5.76 (s, 1H, 2-H), 7.07–7.16 (m, 2H, H_arom._), 7.43–7.51 (m, 2H, H_arom._); ^13^C-NMR (CDCl_3_): δ (ppm) = 14.4 (CH_3_–CH_2_–), 39.8 (C-4), 62.0 (–CH_2_–CH_3_), 99.4 (C-2), 116.5 (d, *J_C-3`, F& C-5`, F_* = 21.95 Hz, C-3` and C-5`), 117.1 (C≡N), 128.6 (d, *J_C-2`, F& C-6`, F_* = 8.57 Hz, C-2` and C-6`), 133.5 (d, *J_C-1`, F_* = 3.82 Hz, C-1`), 155.1 (C-3), 164.4 (d, *J_C-4`, F_* = 252.23 Hz, C-4`), 168.9 (C-1).

*(E)-4-Cyano-3-(3-methoxy-phenyl)-2-butenoic acid ethyl ester* [(*E*)-**3g**]. Yield 53%; IR (neat): ν (cm^−1^) = 2216, 1733, 1599, 1577, 1229, 1177; ^1^H-NMR (CDCl_3_): δ (ppm) = 1.22 (t, *J* = 7.03 Hz, 3H, CH_3_–CH_2_–), 3.84 (s, 3H, OCH_3_), 3.89 (s, 2H, 4-H), 4.16 (q, *J* = 7.03 Hz, 2H, –CH_2_–CH_3_), 5.79 (s, 1H, 2-H), 6.97–7.37 (m, 4H, H_arom._); ^13^C-NMR (CDCl_3_): δ (ppm) = 14.4 (CH_3_–CH_2_–), 39.8 (C-4), 55.8 (OCH_3_), 61.9 (–CH_2_–CH_3_), 99.7 (C-2), 112.4, 116.2 (C_arom._), 117.2 (C≡N), 118.9, 130.5, 138.7 (C_arom._), 156.2 (C-3), 160.3 (C_arom._), 168.9 (C-1).

*(E)-4-Cyano-3-(4-methoxy-phenyl)-2-butenoic acid ethyl ester* [(*E*)-**3h**] [32]. Yield 45%; IR (neat): ν (cm^−1^) = 2213, 1732, 1599, 1514, 1251, 1179; ^1^H-NMR (CDCl_3_): δ (ppm) = 1.21 (t, *J* = 7.03 Hz, 3H, CH_3_–CH_2_–), 3.84 (s, 3H, OCH_3_), 3.88 (s, 2H, 4-H), 4.15 (q, *J* = 7.03 Hz, 2H, –CH_2_–CH_3_), 5.72 (s, 1H, 2-H), 6.92 (d, *J_AB_* = 8.85 Hz, 2H, H_arom._), 7.43 (d, *J_AB_* = 8.85 Hz, 2H, H_arom._). ^13^C-NMR (CDCl_3_): δ (ppm) = 14.4 (CH_3_–CH_2_–), 39.6 (C-4), 55.8 (OCH_3_), 61.9 (–CH_2_–CH_3_), 96.9 (C-2), 117.7 (C≡N), 155.3 (C-3), 114.8, 128.1, 129.4, 161.9 (C_arom._), 169.2 (C-1).

#### 3.1.6. General Procedure for the Preparation of (*R,S*)-5-Amino-3-arylpentanoic Acid Hydrochlorides **1a–h**

To a solution of (*E*)–3-aryl-4-cyano-2-butenoic acid ethyl esters **3a**–**c** and/or **3e**–**h** (2 mmol) in 95% ethanol (10 mL) and concentrated hydrochloric acid (1 mL) was added PtO_2_ (0.05 g) for compounds **3a**, **3b**, **3e** and **3f** or 10% Pd/C (0.10 g) for compounds **3b**, **3c**, **3g** and **3h**. The reaction mixture was hydrogenated on a Parr shaker apparatus under 4 bar of H_2_ for 18 h at room temperature. The catalyst was removed by filtration and the solvent was evaporated under reduced pressure to give (*RS*)-5-amino-3-arylpentanoic acid ethyl ester hydrochlorides **2a**–**h** which were dissolved in 5 N hydrochloric acid (15 mL) and washed with diethyl ether (2 × 10 mL). Without further purification, the aqueous layer was refluxed with stirring for 4 h. The reaction mixture was evaporated under vacuum to give (*RS*)-5-amino-3-aryl-pentanoic acid hydrochlorides **1a**–**h** which were recrystallized from the isopropanol.

*(R,S)-5-Amino-3-(4-chlorophenyl)pentanoic acid hydrochloride* (**1a**). Yield 76% as white solid m.p. 201–203 °C; IR (neat): ν (cm^−1^) = 3200–2727 and 1726; ^1^H-NMR (D_2_O): δ (ppm) = 1.81–2.05 (m, 2H, 4-H), 2.50–2.87 (m, 4H, 2-H and 5-H), 2.99-3.12 (m, 1H, 3-H), 7.17 (d, *J_AB_* = 8.55 Hz, 2H, H_arom._), 7.26 (d, *J_AB_* = 8.55 Hz, 2H, H_arom._); ^13^C-NMR (D_2_O): δ (ppm) = 33.2 (C-4), 38.0 (C-2), 39.1 (C-3), 41.1 (C-5), 129.2, 129.4, 132.7, 140.9 (C_arom._), 176.6 (C-1); MS (EI), m/z (%): 209 (100), 181 (30), 138 (64), 97 (56), 43 (43); MS (CI), m/z (%): 227 [(100), M^+^].: Anal. Calcd. for C_11_H_15_Cl_2_NO_2_: C 50.02, H 5.72, N 5.30; found C 49.93, H 5.72, N 5.36.

*(R,S)-5-Amino-3-(2,4-chlorophenyl)pentanoic acid hydrochloride* (**1b**). Yield 70% as white solid m.p. 215–217 °C; IR (neat): ν (cm^−1^) = 3200–2700 and 1728; ^1^H-NMR (D_2_O): δ (ppm) = 1.87–2.12 (m, 2H, 4-H), 2.57–2.98 (m, 4H, 2-H and 5-H), 3.58–3.70 (m, 1H, 3-H), 7.20–7.32 (m, 3H, H_arom._); ^13^C-NMR (D_2_O): δ (ppm) = 32.6 (C-4), 35.0 (C-2), 37.8 (C-3), 39.7 (C-5), 128.3, 129.3, 129.7, 133.1, 134.6, 138.5 (C_arom._), 176.3 (C-1); MS (EI), m/z (%): 243 (37), 208 (72), 172 (49), 97 (100), 43 (46); MS (CI), m/z (%): 261 [(100), M^+^ -1]; Anal. Calcd. for C_11_H_14_Cl_3_NO_2_: C 44.25, H 4.73, N 4.69; found C 44.10, H 4.76, N 4.79.

*(R,S)-5-Amino-3-(4-methylphenyl)pentanoic acid hydrochloride* (**1c**). Yield 78% as white solid m.p. 204–206 °C; IR (neat): ν (cm^−1^) = 3200-2720 and 1726; ^1^H-NMR (D_2_O): δ (ppm) = 1.80–2.03 (m, 2H, 4-H), 2.19 (s, 3H, 4`-CH_3_), 2.50–2.86 (m, 4H, 2-H and 4-H), 2.96–3.08 (m, 1H, 3-H), 7.12 (s, 4H, H_arom._); ^13^C-NMR (D_2_O): δ (ppm) = 20.5 (4`-CH_3_), 33.3 (C-4), 38.1 (C-2), 39.3 (C-3), 41.3 (C-5), 127.8, 129.9, 137.8, 139.2 (C_arom._), 176.9 (C-1); MS (CI), m/z (%): 207 [(100), M^+^]; Anal. Calcd. for C_12_H_18_ClNO_2_: C 59.14, H 7.44, N 5.75; found C 58.75, H 7.39, N 5.76.

*(R,S)-5-Amino-3-phenylpentanoic acid hydrochloride* (**1d**). Yield 69% as white solid m.p. 195–196 °C; IR (neat): ν (cm^−1^) = 3200–2690 and 1724; ^1^H-NMR (D_2_O): δ (ppm) = 1.83–2.06 (m, 2H, 4-H), 2.54–2.86 (m, 4H, 2-H and 5-H), 3.00–3.12 (m, 1H, 3-H), 7.18–7.33 (m, 5H, H_arom._); ^13^C-NMR (D_2_O): δ (ppm) = 33.3 (C-4), 38.1 (C-2), 39.7 (C-3), 41.2 (C-5), 127.8, 127.9, 129.4, 142.3 (C_arom._), 176.9 (C-1); MS (EI), m/z (%): 194 [(10) M^+^+ 1], 175 (95), 104 (100), 91 (41), 43 (42); Anal. Calcd. for C_11_H_16_ClNO_2_: C 57.52, H 7.02, N 6.09; found C 57.12, H 7.13, N 5.99.

*(R,S)-5-Amino-3-(3,4-chlorophenyl)pentanoic acid hydrochloride* (**1e**). Yield 80% as white solid m.p. 201–203 °C; IR (neat): ν (cm^−1^) = 3200–2700 and 1715; ^1^H-NMR (D_2_O): δ (ppm) = 1.81–2.05 (m, 2H, 4-H), 2.51–2.95 (m, 4H, 2-H and 5-H), 3.00–3.12 (m, 1H, 3-H), 7.09–7.39 (m, 3H, H_arom._); ^13^C-NMR (D_2_O): δ (ppm) = 32.9 (C-4), 37.9 (C-2), 38.9 (C-3), 40.9 (C-5),127.7, 129.8, 130.7, 131.1, 132.4, 142.9 (C_arom._), 176.4 (C-1); MS (CI), m/z (%): 261 [(100), M^+^ -1]; Anal. Calcd. for C_11_H_14_Cl_3_NO_2_: C 44.25, H 4.73, N 4.69; found C 44.04, H 4.99, N 4.72.

*(R,S)-5-Amino-3-(4-fluorophenyl)pentanoic acid hydrochloride* (**1f**). Yield 81% as white solid m.p. 208–210 °C; IR (neat): ν (cm^−1^) = 3200–2700 and 1724. ^1^H-NMR (D_2_O): δ (ppm) = 1.81–2.05 (m, 2H, 4-H), 2.49–2.87 (m, 4H, 2-H and 5-H), 3.00–3.12 (m, 1H, 3-H), 6.96–7.03 (m, 2H, H_arom._), 7.17–7.23 (m, 2H, H_arom._); ^13^C-NMR (D_2_O): δ (ppm) = 33.3 (C-4), 38.0 (C-2), 38.9 (C-3), 41.3 (C-5), 115.9 (d, *J_C-3`, F& C5`, F_* = 21.38 Hz, C-3` and C-5`), 129.5 (d, *J_C-2`, F& C-6`, F_* = 8.17 Hz, C-2` and C-6`), 137.9 (d, *J_C-1`, F_* = 3.02 Hz, C-1`), 162.0 (d, *J_C-4`, F_* = 242.87 Hz, C-4`), 176.8 (C-1); MS (CI), m/z (%): 211 [(100), M^+^]; Anal. Calcd. for C_11_H_15_ClFNO_2_: C 53.34, H 6.10, N 5.66; found C 53.17, H 6.34, N 5.66.

*(R,S)-5-Amino-3-(3-methoxyphenyl)pentanoic acid hydrochloride* (**1g**). Yield 85% as pale yellow solid m.p. 182–184 °C; IR (neat): ν (cm^−1^) = 3200–2700 and 1722; ^1^H-NMR (D_2_O): δ (ppm) = 1.82–2.04 (m, 2H, 4-H), 2.52–2.87 (m, 4H, 2-H and 5-H), 2.98–3.10 (m, 1H, 3-H), 3.69 (s, 3H, OCH_3_), 6.77–7.25 (m, 4H, H_arom._); ^13^C-NMR (D_2_O): δ (ppm) = 33.2 (C-4), 38.1 (C-2), 39.7 (C-3), 41.1 (C-5), 55.7 (OCH_3_), 113.1, 113.6, 120.6, 130.6, 144.2, 159.6 (C_arom._), 176.8 (C-1); MS (CI), m/z (%): 223 [(100), M^+^]; Anal. Calcd. for C_12_H_18_ClNO_3_: C 55.49, H 6.99, N 5.39; found C 55.20, H 7.01, N 5.33.

*(R,S)-5-Amino-3-(4-methoxyphenyl)pentanoic acid hydrochloride* (**1h**). Yield 76% as pale yellow solid m.p. 194–195 °C; (neat): ν (cm^−1^) = 3200–2721 and 1724; ^1^H-NMR (D_2_O): δ (ppm) = 1.79–2.03 (m, 2H, 4-H), 2.49–2.86 (m, 4H, 2-H and 5-H), 2.96–3.08 (m, 1H, 3-H), 3.68 (s, 3H, OCH_3_), 6.86 (d, *J_AB_* = 8.85 Hz, 2H, H_arom._), 7.15 (d, *J_AB_* = 8.85 Hz, 2H, H_arom._). ^13^C-NMR (D_2_O): δ (ppm) = 33.4 (C-4), 38.1 (C-2), 38.9 (C-3), 41.4 (C-5), 55.8 (OCH_3_), 114.7, 129.0, 134.8, 158.2 (C_arom._), 176.9 (C-1); MS (CI), m/z (%): 223 [(100), M^+^]; Anal. Calcd. for C_12_H_18_ClNO_3_: C 55.49, H 6.99, N 5.39; found C 55.23, H 7.07, N 5.35.

#### 3.1.7. Synthesis of Cyanoacetic Acid (**6i**)

A mixture of ethyl cyanoacetate (**7i**, 10 g, 88 mmol) and 1 N hydrochloric acid (35 mL) was heated at 100 °C for 1.5 h. The reaction mixture was evaporated under reduced pressure to give 7.5 g (100%) of **6i** as a colorless crystals m.p. 63–65 °C which was pure enough to be used in the next step without further purification. IR (neat): ν (cm^−1^) = 3300–2973, 2269, 1725, 1388, 1183; ^1^H-NMR (DMSO-*d_6_*): δ (ppm) = 3.28 (s, 2H, 2-H), 8.1–8.7 (br.s, 1H, COOH); ^13^C-NMR (DMSO-*d_6_*): δ (ppm) = 25.5 (C-2), 116.3 (C≡N), 166.5 (C-1).

#### 3.1.8. Synthesis of 4-Cyano-3-methyl-2-butenoic Acid Ethyl Ester (**5i**)

A mixture of cyanoacetic acid (**6i**, 4.51 g, 53 mmol), ethyl acetoacetate (6.51 g, 50 mmol), ammonium acetate (0.77 g, 10 mmol) and acetic acid (1.58 g, 1.5 mL, 26.3 mmol) in benzene (15 mL) was refluxed for 8 h using a Dean-Stark apparatus. The reaction mixture was evaporated under reduced pressure, water (10 mL) was added to the residue and extracted with diethyl ether (3 × 15 mL). The organic layer was separated, dried (Na_2_SO_4_) and evaporated under vacuum. The residue was distilled under vacuum to yield 5.2 g (68%) of **5i** as a colorless oil b.p. 100–102 °C/5 mm (lit. [19] 130 °C/20 mm) with *E*/*Z* ratio = 1.7 as detected by ^1^H-NMR. IR (neat): ν (cm^−1^) = 2221, 1733, 1636, 1175, 1161; ^1^H-NMR (CDCl_3_): δ (ppm) = 1.24–1.31 (2 x t, 3H, CH_3_–CH_2_–), 2.01 [d, *J* = 1.53 Hz, 3H, (*Z*)-3-CH_3_], 2.13 [d, *J* = 0.93 Hz, 3H, (*E*)-3-CH_3_], 3.18 [d, *J* = 0.90 Hz, 2H, (*E*)-4-H], 3.42 [s, 2H, (*Z*)-4-H], 4.12–4.22 (2 × q, 2H, –CH_2_–CH_3_), 5.29–5.32 (m, 1H, 2-H); ^13^C-NMR (CDCl_3_): δ (ppm) = 14.5 (CH_3_–CH_2_–), 21.7 [(*E*)-3-CH_3_], 23.8 [(*Z*)-3-CH_3_], 41.6 [(*Z*)-C-4], 43.9 [(*E*)-C-4], 61.8 (–CH_2_–CH_3_), 99.7 [(*E*)-C-2], 99.8 [(*Z*)-C-2], 116.6 [(*Z*)-C≡N], 116.7 [(*E*)-C≡N], 157.1 [(*Z*)-C-3], 157.2 [(*E*)-C-3], 168.9 [(*Z*)-C-1], 169.2 [(*E*)-C-1].

#### 3.1.9. Synthesis of (*R,S*)-5-Benzyloxycarbonylamino-3-methylpentanoic Acid (**2i**)

To a solution of 4-cyano-3-methyl-2-butenoic acid ethyl ester (**5i**, 0.77 g, 5 mmol) in 95% ethanol (25 mL) was added concentrated hydrochloric acid (1 mL) and 10% Pd/C (0.26 g). The reaction mixture was hydrogenated on a Parr shaker apparatus under 4 bar of H_2_ for 18 h at room temperature. The catalyst was removed by filtration and the solvent was evaporated under vacuum to give (*RS*)-5-amino-3-methyl-pentanoic acid ethyl ester hydrochloride (**4i**) which was dissolved in 5 N hydrochloric acid (10 mL) and extracted with diethyl ether (3 × 10 mL). Without further purification the aqueous layer was refluxed under stirring for 4 h. The reaction mixture containing (*RS*)-5-amino-3-methyl-pentanoic acid hydrochloride (**3i**) was cooled (0–5 °C) and basified using 4 N sodium hydroxide solution (14 mL). To this basic solution was added simultaneously in portions and under cooling (0 °C) benzyl chloroformate (0.85 g, 5 mmol) and 4 N sodium hydroxide solution (1.25 mL) during 30 min. The reaction mixture was extracted with diethyl ether (3 × 10 mL), the aqueous layer was cooled (0–5 °C) and acidified using concentrated hydrochloric acid. The reaction mixture was extracted with diethyl ether (3 × 10 mL), dried (Na_2_SO_4_) and evaporated under reduced pressure to give 0.86 g (65%) of **2i** as a viscous pale yellow oil which was used in the next step without further purification. IR (neat): ν (cm^−1^) = 3066–2588, 1699, 1528, 1454, 1523; ^1^H-NMR (CDCl_3_): δ (ppm) = 1.02 (d, *J* = 6.1 Hz, 3H, 3-CH_3_), 1.39–1.50 (m, 1H, 4-H_a_), 1.53–1.67 (m, 1H, 4-H_b_), 1.98–2.15 (m, 1H, 3-H), 2.21–2.55 (m, 2H, 2-H), 3.25 (m, 2H, 5-H), 5.03 (br.s 1H, N–H), 5.13 (s, 2H, –CH_2_–C_6_H_5_), 7.37 (s, 5H, H_arom._), 10.27 (br.s, 1H, COOH); ^13^C-NMR (CDCl_3_): δ (ppm) = 19.9 (3-CH_3_), 27.9 (C-3), 36.8 (C-4), 39.3 (C-5), 41.7 (C-2), 67.2 (–CH_2_–C_6_H_5_), 127.5, 128.6, 128.9, 136.9 (C_arom._), 157 (O=C–N–H), 178.8 (C-1).

#### 3.1.10. Synthesis of (*R,S*)-5-Amino-3-methylpentanoic Acid (**1i**)

To a solution of (*R,S*)-5-benzyloxycarbonylamino-3-methyl-pentanoic acid (**2i**, 0.53 g, 2 mmol) in 50% 2-propanol (10 mL) was added 10% Pd/C (0.85 g). The reaction mixture was hydrogenated on a Parr shaker apparatus under 4 bar of H_2_ for 18 h at room temperature. The catalyst was removed by filtration and the solvent was evaporated under vacuum. The residue was recrystallized (2-propanol/water) to give 0.18 g (69%) of **1i** as a white powder m.p. 164–165 °C (lit. [21] 133–135 °C). IR (neat): ν (cm^−1^) = 3019–2659, 1630, 1528, 1460, 1398; ^1^H-NMR (D_2_O): δ (ppm) = 0.78 (d, *J* = 6.73 Hz, 3H, 3-CH_3_), 1.31–1.58 (m, 2H, 4-H), 1.71–1.85 (m, 1H, 3-H), 1.87–1.96 (m, 1H, 2-H_a_), 2.02–2.10 (m, 1H, 2-H_b_), 2.77–2.95 (m, 2H, 5-H); ^13^C-NMR (CDCl_3_): δ (ppm) = 19.1 (3-CH_3_), 28.5 (C-3), 33.9 (C-4), 37.9 (C-5), 44.7 (C-2), 181.9 (C-1); MS (CI), m/z (%): 149.1 [(100), M^+^+18]; Anal. Calcd. for C_6_H_13_NO_2_: C 54.94, H 9.99, N 10.68; found C 54.64, H 10.11, N 10.60.

### 3.2. Pharmacological Evaluation

#### 3.2.1. Materials

Culture media, serum and antibiotics were obtained from Invitrogen (Paisley, UK). The rat GABA_B_R plasmids and the Gαq-z5 construct were generous gifts from Dr. Janet Clark (National Institute of Health, Bethesda, MD, USA) and Dr. Bruce Conklin (University of California, San Francisco, CA, USA). The tsA201 cells were a generous gift from Dr. Penelope S. V. Jones (University of California, San Diego, CA, USA).

#### 3.2.2. Methods

TsA201 cells (a transformed human embryonic kidney (HEK) 293 cell line) [33] were maintained at 37 °C in a humidified 5% CO_2_ incubator in Dulbecco’s modified Eagle medium (DMEM) supplemented with penicillin (100 U/mL), streptomycin (100 mg/mL) and 10% fetal calf serum. One million cells were split into a 10 cm tissue culture plate and transfected the following day with 0.7 μg GABA_B_R1b-pcDNA3.1, 3.5 μg GABA_B_R2-pcDNA3.1 and 0.7μg Gαq-z5-pcDNA using SuperFect as a DNA carrier according to the protocol by the manufacturer (Qiagen, Hilden, Germany). The day after transfection, cells were split into one poly-d-lysine coated 96-well black-walled–clear-bottomed tissue culture plates in the same medium as mentioned above and incubated overnight. The following day the measurement of intracellular calcium was performed as follows. The media was exchanged with Hanks balanced saline solution containing 1 mM CaCl_2_, 1 mM MgCl_2_, 20 mM HEPES, 2.5 mM probencid and 4 μM Fluo-4AM (pH = 7.4). The cells were incubated for 1 h at 37 °C in a humidified 5% CO_2_ incubator. Cells were then washed twice with the same buffer without Fluo-4AM and finally 100 μL of the buffer was left in the wells. The cell plate was then transferred to the NovoStar (BMG Labtechnologies, Offenburg, Germany) and the basal fluorescence level was adjusted to ~10,000 fluorescence units (FU) using excitation/emission wavelengths of 485–520 nm, respectively. Fluorescence readings were measured for 45 s after addition of ligand and response was calculated as peak response minus basal level. Inactive compounds were also tested as antagonists. Twenty min after application of ligand, 10 μM GABA was added to the well and fluorescence was measured as above.

#### 3.2.3. Data Analysis

All data analysis has been carried out using GraphPad Prism version 6.0c for Mac OS X (GraphPad Software, San Diego, CA, USA). Concentration-response curves have been fitted by non-linear regression using the equation for sigmoidal concentration-response function:

R = R_min_ + (R_max_ − R_min_)/(1 + 10 ^ (logEC_50_ − X))

in which X is the logarithm of the agonist concentration, R is the response, R_max_ is the maximal response, R_min_ is the minimal response and EC_50_ is the concentration giving half maximum response. All experiments were performed in triplicate and the results are given as mean pEC_50_ ± S.E.M of 3–4 experiments.

## 4. Conclusions

Synthesis and GABA_B_R agonistic activity of certain amino acids **1a**–**i** as homologues of the clinically used drug, baclofen (**1**), are reported. The presence of an aryl moiety in position three of the DAVA backbone is essential for GABA_B_R agonistic activity as replacement of this aryl moiety with a methyl group gave compound **1i** which is devoid of GABA_B_R agonistic activity. Additionally, the substitution pattern of this aryl moiety plays an important role in the exhibited GABA_B_R agonistic activity. Thus, mono-substitution on the aromatic moiety attached to the three position of the DAVA backbone with a halogen, especially *para*-chloro (compound **1a**), is optimum for GABA_B_R agonistic activity. Compound **1a** showed GABA_B_R agonistic activity with EC_50_ = 46 μM, being the most active congener in the whole synthesized series.
